# Draft Genome Sequence of *Acinetobacter johnsonii* MB44, Exhibiting Nematicidal Activity against *Caenorhabditis elegans*

**DOI:** 10.1128/genomeA.01772-15

**Published:** 2016-02-18

**Authors:** Shijing Tian, Muhammad Ali, Li Xie, Lin Li

**Affiliations:** State Key Laboratory of Agricultural Microbiology, Huazhong Agricultural University, Wuhan, China

## Abstract

*Acinetobacter johnsonii* MB44 was isolated from a frost-plant-tissue sample, which showed noteworthy nematicidal activity against the model organism *Caenorhabditis elegans*. Here, we report the 3.4 Mb draft genome of *A. johnsonii* MB44, which will help in understanding the molecular mechanism of its ability to infect nematodes.

## GENOME ANNOUNCEMENT

Bacterial species of the genus *Acinetobacter* are ubiquitous in nature and are usually found in the hospital environment; some of these species have been implicated in a variety of nosocomial infections (1, 2). The genus *Acinetobacter* consisted of 43 validly described species until 2015 (http://www.bacterio.net/acinetobacter.html). *Acinetobacter johnsonii*, which was first proposed and described by Bouvet and Gimont in 1986 (3), has rarely been reported to cause clinical infections (4, 5). We isolated *A. johnsonii* MB44 from a frost-plant-tissue sample in the process of screening for ice-nucleating bacteria in China (6). The strain showed remarkable nematicidal activity against *Caenorhabditis elegans* (unpublished data). To identify the potential nematode-virulent factors, we sequenced and annotated the genome of *A. johnsonii* MB44.

Genomic DNA was extracted using a bacterial DNA kit (GBCBIO), and DNA quantity was determined with a Nanodrop spectrometer (Thermo Scientific, Wilmington, NC, USA). The genome sequencing of *A. johnsonii* MB44 was performed via Illumina HiSeq 2000 platform using the paired-end strategy (2 × 125 bp). A total of 8,593,104 reads with approximately 137-fold coverage were assembled via ABySS version 1.3.7 (7) using a k-mer size of 90. Through the data assembly, 75 contigs with a total length of 3,357,599 bp and an average G+C content of 41.37 were obtained, and the contig *N*_50_ was found to be 106,230 bp.

The annotation of the genome was performed by the NCBI Prokaryotic Genome Annotation Pipeline (http://www.ncbi.nlm.nih.gov/genome/annotation_prok/), which predicted 3,277 genes, including 3,064 coding sequences (CDS), 21 rRNAs, and 81 tRNAs. A total of 58.00% of CDSs could be assigned to the cluster of orthologous groups of proteins (COG) database, and 3 clusters of regularly interspaced short palindromic repeat (CRISPR) repeats were found using CRISPRFinder (8). The 16S rRNA gene sequence of *A. johnsonii* MB44 was 99.65% identical to the type strain *A. johnsonii* ATCC 17909^T^. The average nucleotide identity (ANI) using the online calculator (http://enve-omics.ce.gatech.edu/ani/index) revealed a two-way ANI value of 95.65% between *A. johnsonii* MB44 and *A. johnsonii* ATCC 17909^T^, which suggests that these two strains belong to same speices.

We used the software MP3 (9) to predict virulent proteins in this genomic data and 108 potential virulent proteins were found. In addition, this genome encodes some proteins that are homologous to the known virulent proteins in *A. baumannii*, such as the outer membrane protein A, the phospholipase D, and penicillin-binding protein 7/8 (10). The genome of MB44 also contains genes involved in the biosynthesis of siderophore and capsular polysaccharide, which are important virulence factors associated with the killing of *C. elegans* (11, 12). The genome information of *A. johnsonii* MB44 may accelerate our understanding of the molecular mechanism of its ability to infect nematodes.

### Nucleotide sequence accession numbers.

This whole-genome shotgun project was deposited at DDBJ/EMBL/GenBank under the accession no. LBMO00000000. The version described in this paper is version LBMO01000000.
